# Exploring determinants of time to school re-entry after pediatric epilepsy surgery

**DOI:** 10.1016/j.ebr.2025.100771

**Published:** 2025-04-14

**Authors:** Evangeline A. Huis in 't Veld, Olga Braams, Willem M. Otte, Peter van Rijen, Kees P.J. Braun, Renske Schappin

**Affiliations:** a Department of Pediatric Psychology and Social Work, University Medical Center Utrecht, the Netherlands; b Department of Child Neurology, University Medical Center Utrecht, the Netherlands; c Department of Neurosurgery, University Medical Center Utrecht, the Netherlands; d Department of Child Neurology, University Medical Center Utrecht, Member of ERN EpiCARE, the Netherlands; e Department of Surgery, University Medical Center Utrecht, the Netherlands

**Keywords:** Education, Child, Interview, Counseling, Duration of hospitalization

## Abstract

After epilepsy surgery, it varies when children re-enter school. The aim of this study was to identify determinants for this variation. Parents of 21 school-attending children participated in semi-structured interviews during their child’s hospitalization for epilepsy surgery and one year afterward (based on the standard neuropsychological post-surgical follow-up). The mean time to school re-entry was 10.7 weeks (SD = 6.3). One child did not attend school after one year, whilst the fastest child resumed school 2 weeks after surgery. We performed univariable linear regression models with bootstrapped R^2^ for all variables deemed theoretically or clinically relevant to school re-entry. We found that temporal surgery was significantly related to shorter time to school re-entry; and that longer hospitalization, and presurgical outpatient educational counseling were significantly related to longer time to school re-entry. In multivariable linear regression, these variables together predicted 57% of variance (bootstrapped) in time to school re-entry. In conclusion, our findings indicate that school re-entry varies considerably among children but can partly be explained by surgery related variables and the presence of counseling. Given the importance of school in children’s daily lives, we argue that school attendance should be stimulated by increasing professionals’ awareness of children’s school re-entry process.

## 1 Introduction

Education is of pivotal importance in children’s daily life and contributes to both cognitive and social-emotional development [1]. Children spend 57% of their waking hours in school [2]. Yet, children with epilepsy face numerous barriers to attending school. Previous studies have revealed that children with epilepsy are at increased risk of educational underachievement, learning difficulties, social isolation, and poor self-esteem [3], [4]. For children with refractory epilepsy, surgery is considered an effective treatment since it can lead to both seizure and medication freedom [5]. Although cognitive decline may occur shortly after surgery [6], in the long-term, pediatric epilepsy surgery may prevent this decline or even improve cognitive functioning [5]. Therefore, epilepsy surgery may positively influence school performance. A previously published report on the long-term employment outcomes of adolescents who underwent epilepsy surgery in their childhood revealed a positive impact of pediatric epilepsy surgery as well [7]. Given the importance of school and the proposed positive effect of epilepsy surgery on cognitive functioning, we explored determinants of time to school re-entry after epilepsy surgery. Knowledge about these factors could improve child and parent counseling on children’s school careers after surgery.

## 2 Methods

### 2.1 Participants

Parents of school-aged children who underwent epilepsy surgery between April 2016 and July 2017 at the University Medical Center Utrecht (UMCU) were invited to participate in this study. The only inclusion criterion was that the child attended formal education. Parents were requested to participate in a semi-structured interview during their child’s hospitalization for epilepsy surgery. Secondary school-aged children were asked to participate as well. Approximately a year after surgery, participants were approached again for a second interview by phone. The complete semi-structured interviews used in the first and second assessments are shown in Supplementary Information S1 and Supplementary Information S2, respectively. The Institutional Review Board of the UMCU stated that the Dutch Law on Medical Research Involving Human Subjects did not apply to this study.

### 2.2 Measures

Cognitive functioning and illness variables were retrieved from medical records. Due to the small sample size, we classified the brain area of surgery into three categories: 1) temporal, 2) extratemporal, or 3) both (temporal combined with extratemporal), following Ochoa-Escudero and colleagues [8].

The semi-structured interviews consisted of open and closed questions, and were designed by the authors. The first interview addressed demographic and general school information such as the child’s school career, parents’ reasons for choosing a specific school for their child, parents’ perceptions of how the school handled their child’s epilepsy, and the upcoming surgery. In addition to the first interview, the second interview covered surgical outcomes, the child’s school career after surgery, and the support received from both the hospital and school regarding school re-entry. Parents were also asked how many weeks it took their child to fully resume school (i.e., attending the same hours per week as before surgery), measured from the day of surgery.

### 2.3 Data management and analysis

To enable quantitative analysis of parents’ answers, we dichotomized two closed questions and three semi-open questions (yes vs. no). Classification was performed by the first author, in consensus with the second and last author.

Although time to school re-entry could be considered time-to-event data, its distribution was normal with a long right tail. Therefore, we performed regression analysis. Given the small sample size, we examined univariable linear regression models with bootstrapped (2500 samples with replacement) R^2^ (95 % CI) for all variables deemed theoretically or clinically relevant to school re-entry.

Variables that were univariably significant with p < 0.05 were included in a multivariable regression with bootstrapped R^2^.

Investigated variables were: age at first interview, the Full Scale Intelligence Quotient (FSIQ) from presurgical age-adapted cognitive test sets, length of hospitalization (in days, from day of surgery to day of discharge), postsurgical seizure freedom (no seizure after surgery at all), grid implantation, brain area of surgery, presurgical educational level (regular vs. special), presurgical presence of outpatient educational counseling (for an explanation of the function of an educational counselor, see Supplementary Information S3), presurgical enjoyment in school, whether parents deemed school results of importance after surgery, and parental educational level (low and middle vs. high) [9]. A p-value of < 0.05 was considered statistically significant. Effect size was measured using Cohen’s R^2^, with thresholds of R^2^ ≥ 0.01 for small, R^2^ ≥ 0.09 for medium, and R^2^ ≥ 0.25 for large effects [10]. Analyses were performed using R software [11].

## 3 Results

### 3.1 Participants

We invited the parents of 25 children to participate in this study; all consented and completed the first interview. None of the secondary school-aged children did choose to participate, however their parents did. Parents of 21 children also participated in the second interview. Additionally, the parents of three children were contacted in an extra follow-up round after the second interview to data on time to school re-entry, which was unavailable at the time of the second interview. The parents of three children could not be reached despite repeated attempts, and one parent was unable to participate due to family circumstances. One child took 50 weeks to return to school. According to the parents, this delay was due to family circumstances at the time and was unrelated to illness or surgery. Therefore, this case was excluded from the outcome measure.

The four children who dropped out did not differ significantly from the rest of the sample on demographics or illness characteristics. Because time to school re-entry was only available when parents participated in the second interview, we present data on the 21 children whose parents participated in both interviews (Table 1). All functional outcomes for the 19 children included in the analysis are presented in Supplementary Information S4.

**Table 1:** Demographic and illness characteristics of children and educational level of their parents (n = 21).

	N (%) or median (IQR)
Girls	8 (38 %)
Proficient in Dutch	19 (100 %)
Age at first interview, years	12.0 (6.5), range 4–16
Age at epilepsy onset, years	4.5 (10.0), range 0–15
Presurgical FSIQ	75.0 (21.0), range 55–118
Length of hospitalization, days	6.0 (2.0), range 3–17
Etiology	
Tumor	7 (33 %)
MCD	7 (33 %)
Other	7 (33 %)
Brain area of surgery	
Temporal only	6 (29 %)
Extratemporal only	12 (57 %)
Both temporal and extratemporal	3 (14 %)
Hemispherotomy	2 (9 %)
Lesionectomy	1 (5 %)
Grid implantation	3 (14 %)
Postsurgical seizure status (at 1 year)	17 (81 %)
Presurgical child’s educational level	
Regular education	15 (71 %)
Special education	6 (29 %)
Paternal educational level	
Low	2 (10 %)
Middle	10 (48 %)
High	7 (33 %)
Missing	2 (9 %)
Maternal educational level	
Low	3 (14 %)
Middle	11 (52 %)
High	6 (29 %)
Missing	1 (5 %)
Presurgical outpatient educational counseling	10 (48 %)
Presurgical enjoyment in school	17 (81 %)
Parent deemed postoperative school results important	4 (19 %)

Abbreviations: IQR = interquartile range, MCD = malformation of cortical development, FSIQ = Full Scale Intelligence Quotient.

Time to school re-entry followed a normal distribution with a mean of 10.7 weeks (SD = 6.3). The shortest period to school attendance at the presurgical level was 2 weeks. One child never resumed formal education after surgery. While the procedure reduced seizure frequency, it also led to expected neurological deficits, resulting in increased cognitive and behavioral problems. Another child took 50 weeks to resume school, which was not related to epilepsy surgery but to family problems. Neither case was included in the analyses; the first had no measurable outcome and the second was an extreme outlier, leaving 19 children for analysis.

All children remained at the same educational level after surgery, except for one who transitioned from regular to special education within a year. Outpatient educational counseling was discontinued for most children after surgery. However, three continued to receive educational counseling and one newly started it.

### 3.2 Predictors of time to school re-entry after surgery

Univariable analyses are presented in Table 2. All models met the assumptions for linear regression. Children who underwent temporal resection returned to school significantly faster than those with other types of resections. However, due to the small sample size, we were unable to compare the effects of left versus right hemisphere resections. Longer hospital admissions were associated with a delayed return to school. Additionally, children who received outpatient educational counselling before surgery tended to take longer to return to school. Although significance depends on sample size, the bootstrapped effect sizes for these findings were (near) large, indicating they may reflect true effects in the population. We observed a non-significant medium effect for presurgical FSIQ, indicating that higher IQ was associated with a longer time to school re-entry.

**Table 2:** Univariable linear regression models with bootstrapped R^2^ of time to school re-entry in weeks (n = 19).

	B	95 % CI	p-value	R^2^*	95 % CI*
Age at first interview	0.21	−0.61 to 1.03	0.596	0.04	0.00 – 0.43
Presurgical FSIQ	0.08	−0.06 to 0.23	0.255	0.09	0.00 – 0.47
Brain area of surgery					
Temporal only vs. other	−7.51	−13.46 to −1.57	0.016	0.30	0.08 – 0.62
Extratemporal only vs. other	5.16	−0.58 to 10.9	0.075	0.20	0.00 – 0.75
Grid implantation	1.97	−8.14 to 12.08	0.686	0.03	0.00 – 0.29
Hospitalization (days)	0.76	0.05 – 1.46	0.037	0.25	0.00 – 0.68
Postsurgical seizure freedom	1.27	−7.26 to 9.80	0.757	0.01	0.00 – 0.10
Presurgically in special education	−1.56	−8.23 to 5.10	0.627	0.05	0.00 – 0.43
Presurgical enjoyment in school	−0.71	−9.25 to 7.84	0.863	0.01	0.00 – 0.18
Presurgical outpatient educational counseling	5.62	0.08 – 11.17	0.047	0.23	0.01 – 0.58
Postsurgical parent deems school results important	5.32	−4.47 to 15.11	0.267	0.08	0.01 – 0.38
High maternal education	1.92	−3.67 to 7.51	0.478	0.04	0.00 – 0.31
High paternal education	−0.99	−6.46 to 4.48	0.707	0.03	0.00 – 0.32

* Bootstrapped (2500 samples with replacement).

## 4 Multivariable analysis

The bootstrapped R^2^ of the multivariable model was 0.57 (95% confidence interval [CI] 0.26 – 0.83), indicating that the model explained 57% of the variance in time to school re-entry, see Supplementary Information S5. In the multivariable model, none of the predictors were statistically significant (temporal surgery: p = 0.112, hospitalization: p = 0.108, presurgical outpatient counseling: p = 0.232). Given the large proportion of explained variance in the total model, the lack of statistical significance is likely due to the small sample size.

## 5 Discussion

Exploring determinants of school re-entry after epilepsy surgery, we found that the brain area of surgery, duration of hospitalization, and presurgical outpatient educational counseling could be important factors.

Children who underwent temporal resections tended to have shorter hospitalization periods and resumed school more quickly. This may be due to the standardized nature of temporal resections, with less chance of complications and faster postsurgical recovery. Duration of hospitalization can be considered as a measure of the impact of surgery as parents by example reported: *”Our child wanted to re-enter school immediately after surgery but we did not think that was fair to do”*. Children who require more time to recover from surgery or experience postsurgical cognitive or functional difficulties may take longer to resume school. One might expect that assistance of an outpatient educational counselor would accelerate the process of school re-entry, as it can relieve some of the burden of parents, who otherwise take on an educational role themselves: *“We (parents) acted like school and give him a lot of remedial teaching whereby he did not get a backlog in school”.* However, our data did not confirm this hypothesis. Our data is limited by the small and heterogeneous group which prevents us from fully assessing (indirect) mechanisms behind the role of educational counselors. Furthermore, perspectives from other involved professionals (e.g., school staff) regarding school re-entry were not available. More intensive follow-up of these children and future patients will enable more extensive analyses with larger samples, improving our understanding of the mechanisms influencing school re-entry, including neurological factors.

In conclusion, our findings indicate that time to school re-entry varies considerably among children, with surgery-related factors explaining part of this variation. Given the importance of school in children’s daily lives, we argue that school attendance should be actively encouraged by increasing professionals’ awareness of the school re-entry process. Collaboration between parents, schools, counselors, and medical professionals is highly important in supporting children’s return to school.

## CRediT authorship contribution statement

**Evangeline A. Huis in 't Veld:** Writing – original draft, Methodology, Conceptualization. **Olga Braams:** Writing – original draft, Methodology, Conceptualization. **Willem M. Otte:** Writing – review & editing, Methodology, Formal analysis, Conceptualization. **Peter van Rijen:** Writing – original draft, Methodology, Conceptualization. **Kees P.J. Braun:** Writing – review & editing, Supervision, Methodology, Conceptualization. **Renske Schappin:** Writing – review & editing, Writing – original draft, Supervision, Methodology, Formal analysis, Conceptualization.

## Declaration of competing interest

The authors declare that they have no known competing financial interests or personal relationships that could have appeared to influence the work reported in this paper.
