# Supplementary material for: Exploring determinants of time to school re-entry after pediatric epilepsy surgery
Source: Epilepsy Behav Rep. 2025 Apr 14;30:100771. doi: 10.1016/j.ebr.2025.100771 (PMC12168354; doi:10.1016/j.ebr.2025.100771)
Supplement: Supplementary Data 1 [file mmc1.docx]

Supporting information - Exploring determinants of time to school re-entry after pediatric epilepsy surgery

Evangeline A. Huis in ‘t Veld^a,^, Olga Braams^a^, Willem M. Otte^b^, Peter van Rijen^c^, Kees P.J. Braun^d^, Renske Schappin^e^

**Affiliations**

^a^Department of Pediatric Psychology and Social Work, University Medical Center Utrecht, the Netherlands

^b^Department of Child Neurology, University Medical Center Utrecht, the Netherlands;

^c^Department of Neurosurgery, University Medical Center Utrecht, the Netherlands

^d^Department of Child Neurology, University Medical Center Utrecht, member of ERN EpiCARE, the Netherlands

^e^Department of Surgery, University Medical Center Utrecht, the Netherlands. Current affiliation: Department of Dermatology,ErasmusMC/Sophia Children’s Hospital Rotterdam, the Netherlands

**Email addresses**

Evangeline A Huis in ’t Veld: [e.a.huisintveld-2@prinsesmaximacentrum.nl](mailto:e.a.huisintveld-2@prinsesmaximacentrum.nl)

Olga Braams: o.b.braams@umcutrecht.nl

Willem M. Otte: w.m.otte@umcutrecht.nl

Peter van Rijen: p.v.rijen@umcutrecht.nl

Kees P.J. Braun: k.braun@umcutrecht.nl

Renske Schappin: r.schappin@uu.nl

**The corresponding author:**

Correspondence to: Evangeline Huis in ‘t Veld, Wilhelmina Children’s Hospital, University Medical Center Utrecht; email: e.a.huisintveld-2@prinsesmaximacentrum.nl, phone: +31 88 755 5555.

**Supporting materials**

**Supplementary Information S1. Interview 1: During hospitalization for epilepsy surgery**

1. Which type of education is your child following?
2. In which grade is your child?
3. When the answer at q1 = special education: was your child following regular education before?
4. When the answer at q3 = yes, why did your child change from type of education?
5. At which age did your child start with education?
6. Why did you chose this school?
7. Does your child like school? Why?
8. Are there any problems at school?
9. Does your child need supportive care (by example Remedial Teaching)?
10. Did your child repeat a grade?
11. How much importance do you attach to the school performances of your child?
12. How much importance does your child attach to its school performances? How do you notice?
13. Does your child need special care? What care?
14. How do you rate the disease related care at school?
15. Who are the contact persons at school?
16. In which way is the school team informed about the care your child needs?
17. Is an outpatient educational counselor involved in school?
18. Are classmates aware of the epilepsy?
19. How was the class informed about the epilepsy? How did they handle the message of epilepsy?
20. What is the status of your child in class?
21. Does the child have friends? How many true friends?
22. Does your child have behavioral or emotional problems which can be a problem at school?
23. How does your child feel about the upcoming surgery?
24. Do you feel tension in the daily contact with your child?
25. Are there any other problems which can be a problem at school?
26. Did you have adequate contact with your child’s teachers? Why?
27. Do you feel sufficiently heard by school?
28. Does the school inform you sufficiently about your child’s wellbeing?
29. Do you feel like you are actively involved with the school?
30. Does your child have siblings? Are they in the same school?
31. Are the siblings’ school performances affected by the epilepsy?
32. Is the siblings’ behavior affected by the epilepsy?
33. Do you have questions about school at the moment or do you need guidance at school?
34. Any remarks about your child’s education?
35. Any other remarks?

**Supplementary Information S2. Interview 2: One year after surgery**

1. How do you rate the success of the surgery?
2. How did the surgery go?
3. Is your child free of seizures?
4. Does your child still use antiepileptic drugs?
5. How many full days did your child need to re-enter school after surgery?
6. How many halfdays did your child need to re-enter school after surgery?
7. Are there any problems in school at the moment?
8. Did your child repeat a grade after surgery?
9. Did your child change school after surgery?
10. Does your child need any supportive care?
11. Did your child need this care already before surgery or is this new care?
12. Did you need any supportive care or guidance in the past year?
13. Did you receive the needed care or guidance? In which way?
14. Was this guidance adequate?
15. What would you like to change concerning the received guidance?
16. How much importance do you attach to the school performances of your child?
17. Did this change compared to pre-surgery?
18. How much importance does your child attach to its school performances? How do you notice?
19. Has your child’s behavior changed since surgery?
20. Does your child need special care concerning its behavior? Which care?
21. How do you rate the disease related care at school?
22. Is an outpatient educational counselor involved at school?
23. Who are the contact persons at school?
24. How did the class act when your child re-entered school after surgery?
25. How does your child feel about surgery now?
26. What is your child’s status in class since surgery?
27. When the child has siblings:
28. Did the siblings’ behavior change after surgery?
29. Did the siblings’ school performances change after surgery?
30. Do you have questions about school at the moment or do you need guidance at school?
31. Any remarks?
32. Concluding: what changed in the past year compared to life before surgery?

**Supplementary Information S3. Definition of outpatient educational counseling**

Outpatient educational counselors advise and guide (parents of) children and young people with epilepsy in the Netherlands on all types of (special) education.

A targeted approach is defined together with the teacher(s), student support officer, school principal, medical professionals, and the student and his/her parents. In the Netherlands, the national working group on education and epilepsy (*Landelijk Werkverband Onderwijs en Epilepsie*) is a well-known organization that houses outpatient educational counselors. This working group is responsible for sharing advice and expertise in the field of epilepsy and related disorders with schools in the Netherlands. Their aim is to make education more inclusive for students with epilepsy and to build knowledge and expertise. Counselors get normally involved as soon as school-aged children are diagnosed with epilepsy. In the current study, 10/21 children were assisted by an outpatient educational counselor. Data on the reason of the absence of counselors in 11 children was not available.

**Supplementary Information S4.** **Summary of School Re-Entry and Functional Outcomes Post-Surgery**

| PID | Age at surgery (in years) | Surgery type* | Hospitalization (in days) | Complications post | School pre** | School post** | TIQ pre | TIQ post | Counselor pre | Counselor post | Seizure post | Use of anti-epileptic drugs post | School return (in weeks) |
| --- | --- | --- | --- | --- | --- | --- | --- | --- | --- | --- | --- | --- | --- |
| 1 | 11 | 1 | 6 | No | 1 | 4 | 86 | *-* | Yes | Yes | No | Yes | 15 |
| 2 | 16 | 1 | 3 | No | 4 | 4 | 83 | 83 | Yes | No | No | No | 15 |
| 3 | 14 | 2 | 6 | No | 5 | 5 | 71 | - | Yes | No | Yes | Yes | 8 |
| 4 | 9 | 1 | 6 | No | 1 | 1 | 76 | - | Yes | No | Yes | Yes | 10 |
| 5 | 14 | 1 | 6 | No | 4 | 4 | 83 | - | Yes | No | No | Yes | 18 |
| 6 | 14 | 1 | 7 | No | 4 | 4 | 64 | - | Yes | Yes | No | No | 16 |
| 7 | 16 | 1 | 17 | Yes | 4 | 4 | 116 | - | Yes | Yes | No | No | 16 |
| 8 | 7 | 1 | 5 | No | 1 | 2 | 62 | - | Yes | No | No | No | 7 |
| 9 | 4 | 0 | 5 | No | 1 | 1 | 74 | - | Yes | No | No | No | 3 |
| 10 | 7 | 2 | 18 | No | 2 | 2 | - | - | Yes | No | No | No | 26 |
| 11 | 7 | 1 | 6 | No | 1 | 1 | 96 | - | No | No | No | No | 17 |
| 12 | 16 | 1 | 5 | No | 4 | 6 | 83 | 83 | No | No | No | No | 8 |
| 13 | 8 | 0 | 7 | No | 1 | 1 | 118 | 111 | No | No | No | No | 6 |
| 14 | 11 | 0 | 5 | No | 1 | 4 | 62 | 66 | No | No | No | No | 8 |
| 15 | 16 | 0 | 5 | No | 4 | 4 | 100 | - | No | No | No | No | 7 |
| 16 | 13 | 1 | 8 | No | 3 | 3 | 55 | 55 | No | No | Yes | No | 11 |
| 17 | 8 | 2 | 11 | No | 2 | 2 | 65 | 65 | No | Yes | No | Yes | 2 |
| 18 | 7 | 1 | 5 | No | 2 | 2 | 71 | 68 | No | No | No | No | 9 |
| 19 | 13 | 0 | 5 | No | 2 | 2 | 65 | - | No | No | No | No | 2 |

*Abbreviations:* PID = Patient ID; Pre = pre-operative; Post = post-operative; TIQ = Total Intelligence Quotient; - = unknown.

*Surgery type categorized in: 0 = temporal surgery; 1 = extra-temporal surgery; 2 = both temporal and extra-temporal surgery.

** School level categorized in: 1 = regular primary education; 2 = special primary education***; 3 = special education***; 4 = regular secondary education; 5 = special secondary education; 6 = postsecondary education.

*** Both special primary education (SBO) and special education (SO) are designed for children in primary school, with SBO providing support for children with mild to moderate learning difficulties, while SO caters to those with more severe disabilities requiring intensive, specialized support.

*Notes:*

- The relatively large amount of missing data for the outcome TIQ is due to the fact that many children had not yet been scheduled for the postoperative intelligence assessment, which is standard of care following pediatric epilepsy surgery, at the time of the second measurement.
- We omitted the variable 'sex' well-considered as including it would pose a risk of identifiability and traceability, given the small and heterogenous sample size of our studygroup.

**Supplementary Information S5. Multivariable linear regression model with bootstrapped R2 of time to school re-entry in weeks (n = 19).**

|  | B | 95% CI | p-value |
| --- | --- | --- | --- |
| Temporal only surgery vs. other | -4.88 | -11.04 – 1.28 | .112 |
| Hospitalization (days) | 0.53 | -0.13 – 1.18 | .108 |
| Presurgical outpatient educational counseling | -3.12 | -8.46 – 2.22 | .232 |

Note. Model bootstrapped R^2^ = 0.57, 95% CI = 0.26 – 0.83.
